# Evaluation of the Modified DECAF Score in Risk Stratification of AECOPD Patients: A Comparative Analysis With the Original DECAF Score

**DOI:** 10.7759/cureus.90194

**Published:** 2025-08-15

**Authors:** Pavithra C, Elen Ann Abraham, Ghanshyam Verma, Ragavi Elango, Aldrin Santhosh

**Affiliations:** 1 Department of Respiratory Medicine, Sree Balaji Medical College and Hospital, Chennai, IND

**Keywords:** acute exacerbation of copd, decaf score, modified decaf score, prognostic tools, risk stratification

## Abstract

Background

Acute exacerbations of chronic obstructive pulmonary disease (AECOPD) significantly contribute to morbidity and mortality worldwide. The DECAF score is a widely used tool for predicting in-hospital mortality in AECOPD patients. A modified Dyspnea, Eosinopenia, Consolidation, Acidemia, and Atrial Fibrillation (DECAF) score incorporating exacerbation frequency has been proposed to enhance prognostic accuracy. This study aims to evaluate the correlation and agreement between the DECAF and modified DECAF scores in assessing severity among AECOPD patients.

Methods

This cross-sectional observational study was conducted at Sree Balaji Medical College and Hospital over one year (January-December 2024). Fifty-one (n = 51) patients admitted with AECOPD were retrospectively analyzed. DECAF and modified DECAF scores were calculated for each patient. Spearman’s rank correlation was used to assess the relationship between the scores. Cohen’s Kappa and McNemar’s test were applied to evaluate agreement and classification shifts.

Results

A strong positive correlation was observed between the DECAF and modified DECAF scores (ρ = 0.702, p < 0.00001). Using the DECAF score, 47 patients (92.2%) were classified as low risk and four patients (7.8%) as high risk, while the modified DECAF reclassified four of the initial low-risk patients into the high-risk category based on exacerbation frequency. However, Cohen’s Kappa showed no agreement beyond chance (κ = 0.00) in risk categorization, and McNemar’s test indicated that this reclassification was not statistically significant (p = 0.125). The lack of agreement may indicate that the modified DECAF’s emphasis on exacerbation frequency identifies different patient profiles, but it could also reflect limitations in its risk stratification ability or be due to the small sample size.

Conclusion

The modified DECAF score demonstrated strong correlation with the original DECAF score and identified additional high-risk patients based on exacerbation history. Recognizing these patients is clinically relevant, as higher modified DECAF scores have been associated with increased mortality, greater need for ventilatory support, and longer hospital stays, factors that can guide decisions about monitoring intensity and resource allocation. However, within our study cohort, no specific treatment or management decisions (such as escalation of care or intervention) were made solely on the basis of risk reclassification by the modified DECAF score. Thus, while the modified DECAF may improve clinician awareness of patients with more unstable disease profiles, further studies are warranted to determine whether its use should prompt tailored treatment strategies or changes in clinical pathways.

## Introduction

Acute exacerbations of chronic obstructive pulmonary disease (AECOPD) represent critical events that significantly impact disease progression, quality of life, hospitalization rates, and mortality. Accurately predicting the severity and outcome of these exacerbations is vital for guiding clinical decision-making and resource allocation. Among the available prognostic tools, the DECAF score, comprising Dyspnea, Eosinopenia, Consolidation, Acidemia, and Atrial Fibrillation, has emerged as a simple yet powerful model for predicting in-hospital mortality in AECOPD patients [1]. Meta-analyses have confirmed the DECAF score’s reliability across varied populations, reinforcing its use in everyday clinical settings to stratify patients by risk [2]. However, ongoing efforts to improve the score's sensitivity and applicability have led to the development of Modified DECAF scores, which attempt to address limitations or tailor the scoring system to different cohorts. These modifications aim to preserve the score’s predictive power while enhancing its usability and alignment with local epidemiological and healthcare dynamics.

Alternative indices, such as the CODEX score, have also been used to evaluate COPD severity, particularly in outpatient settings, by incorporating comorbidities and previous hospitalizations [3]. However, integrated care approaches are often challenged by the overlapping burden of cardiovascular diseases, which are highly prevalent in COPD patients and independently contribute to increased mortality [4]. Additionally, emerging evidence highlights the role of lung microbiota dysbiosis and systemic inflammatory responses in influencing exacerbation severity and frequency. These biological factors, in turn, may affect the prognostic accuracy of scoring systems that rely solely on clinical parameters [5]. Other important contributors to mortality include comorbidities and pulmonary hyperinflation, especially in advanced stages of COPD [6].

Recent perspectives encourage viewing AECOPD as a distinct syndrome rather than a mere worsening of symptoms, prompting more comprehensive phenotypic evaluations and stratified treatment approaches [7]. The Modified DECAF score, as described in prior studies, incorporates exacerbation frequency in addition to the original DECAF components to potentially improve prognostic accuracy in acute exacerbations of chronic obstructive pulmonary disease (AECOPD) patients [8]. The objective of this study was to determine the correlation and agreement between the original and Modified DECAF scores for predicting risk stratification among patients hospitalized with AECOPD. We specifically aimed to compare the proportion of patients classified as high risk by each scoring system and to assess whether the Modified DECAF provides added prognostic value. We hypothesized that the modified DECAF score would identify additional high-risk patients compared to the original DECAF score, owing to the inclusion of exacerbation frequency as an additional parameter, and that this might improve sensitivity for assessing risk in AECOPD.

## Materials and methods

Study design and setting

This cross-sectional observational study was conducted in the Department of Pulmonology, Sree Balaji Medical College and Hospital, between January and December 2024. The study comprised 51 patients admitted with AECOPD. Ethical approval and exemption were granted by the Institutional Review Board of Sree Balaji Medical College and Hospital (IRB Exemption Number: SBMCH/IRB/2024/014) for retrospective, anonymized patient records.

Inclusion and exclusion criteria

All adults (≥18 years) admitted with a confirmed diagnosis of AECOPD were screened. Patients were excluded if they had another chronic respiratory disease as the primary diagnosis, incomplete records relevant to DECAF scoring, or received end-of-life or palliative care. Common comorbidities (including cardiac disease) were not excluded unless they would confound assessment or constitute the primary reason for admission. Full criteria are provided in Table 1.

**Table 1:** Inclusion and Exclusion Criteria for Study Participants The table summarizes the eligibility criteria used for screening patients with acute exacerbation of chronic obstructive pulmonary disease (AECOPD) for the study. Inclusion criteria ensured that participants were appropriate for Dyspnea, Eosinopenia, Consolidation, Acidemia, and Atrial Fibrillation (DECAF) and modified DECAF scoring, while exclusion criteria eliminated confounding chronic respiratory conditions, incomplete records, and end-of-life care cases. Patients with co-existing comorbidities, including cardiac disease, were not excluded unless these conditions constituted a primary diagnosis, confounding chronic respiratory disorder, or resulted in incomplete assessment records.

Category	
Inclusion Criteria	Age ≥ 40 years; Confirmed diagnosis of COPD based on GOLD (Global Initiative for Chronic Obstructive Lung Disease) guidelines; Hospital admission due to AECOPD, defined as an acute worsening of respiratory symptoms requiring additional medical treatment; Availability of all clinical and laboratory parameters necessary to compute both DECAF and Modified DECAF scores
Exclusion Criteria	Diagnosed with other chronic respiratory conditions such as asthma, bronchiectasis, or interstitial lung disease; Incomplete clinical records or missing laboratory values; Terminal illness, palliative care, or patients with documented Do Not Resuscitate (DNR) orders

Definition and verification of exacerbation

An AECOPD exacerbation was defined as a worsening of respiratory symptoms requiring therapy change. Exacerbation frequency was recorded as two or more acute episodes requiring medical attention within the past 12 months. This was verified through review of electronic medical records, hospital discharge summaries, clinic notes, and pharmacy records for corticosteroid or antibiotic use. Where possible, two independent, outcome-blinded investigators cross-validated the exacerbation history to reduce information bias. Patients’ prior acute care episodes were classified by: Number of hospital admissions for AECOPD in the preceding year, ICU admissions, need for intubation, and OPD management with oral corticosteroids or antibiotics. Uncertainties were resolved by consensus. No formal prospective validation of exacerbation data was done, which is acknowledged as a limitation.

Data extraction and rater assignment

Two investigators independently extracted anonymized data using a standardized form. Raters were blinded to in-hospital outcomes during scoring.

Scoring methodology

The DECAF score, comprising five parameters (dyspnea severity by modified Medical Research Council (mMRC) scale, eosinopenia, radiological consolidation, acidemia, and atrial fibrillation), was calculated for each patient following the validated method. Table 2 details the scoring system. The Modified DECAF score retained all original parameters and their weights, with the addition of one point for patients experiencing two or more exacerbations requiring medical attention in the previous year, as per recent literature. The modified DECAF score employed in this research is an academic adaptation conceptualized by the authors for exploratory purposes, with no claims of validation or commercial application.

**Table 2:** The DECAF Scoring System for Predicting In-Hospital Mortality in AECOPD *Modified Medical Research Council (mMRC) Dyspnea Scale. DECAF Score Total: 0–6 points (higher scores indicate greater risk of in-hospital mortality) AECOPD: Acute exacerbation of chronic obstructive pulmonary disease

DECAF Component	Criteria/Value	Points Assigned
Dyspnea (mMRC)*	Grade 5 (too breathless to leave house or breathless when dressing/undressing)	2
	Grade 4 (stops for breath after walking about 100 m or after a few minutes on level ground)	1
	Grade 1-3	0
Eosinopenia	Eosinophil count <0.05 *10^9^/L	1
Consolidation	Radiographic evidence of consolidation	1
Acidemia	Arterial pH <7.30	1
Fibrillation (Atrial)	Presence of atrial fibrillation (on ECG or chart)	1

Statistical analysis

All analyses were performed using IBM SPSS Statistics for Windows, Version 25 (Released 2017; IBM Corp., Armonk, New York, United States). Continuous variables are presented as mean ± standard deviation and categorical data as counts and percentages. Spearman’s rank correlation assessed the association between DECAF and Modified DECAF scores. The chi-square test was used for categorical comparisons. Agreement between DECAF and modified DECAF was further quantified by Bland-Altman analysis; Cohen’s Kappa was used for categorical agreement, noting its limitations with skewed data.

Records with missing or incomplete data were excluded. No formal sample size or power calculation was done due to an exploratory, retrospective design and modest sample; this is a noted limitation.

Ethical considerations

Study exemption was granted by the IRB for the use of anonymized retrospective data. All patient information was kept confidential throughout.

## Results

A total of 51 eligible patients with complete clinical data were included in this retrospective analysis from a single tertiary hospital diagnosed with acute exacerbation of chronic obstructive pulmonary disease (AECOPD) were evaluated using both the DECAF and Modified DECAF scoring systems.

Score distribution

The original DECAF scoring system classified 47 patients (92.2%) as score 0, indicating low severity, and four patients (7.8%) as score 1. None of the patients exceeded a score of 1. In contrast, the Modified DECAF score redistributed several patients: only five patients (9.8%) remained in score 0, while 42 patients (82.4%) were assigned score 1, and four patients (7.8%) were classified as score 2 (Table 3). The chi-square test of independence showed no significant difference in categorical risk assignment between the DECAF and Modified DECAF scoring systems (χ²(2, N = 51) = 2.29, p = 0.130). The effect size, measured using Cramer’s V (V = 0.211), suggests a small association between the two scoring systems.

**Table 3:** Frequency and Percentage Distribution of DECAF and Modified DECAF Scores Among Patients With Acute Exacerbation of COPD (AECOPD) Frequency and percentage distribution of Dyspnea, Eosinopenia, Consolidation, Acidemia, and Atrial Fibrillation (DECAF) and Modified DECAF scores among patients with acute exacerbation of chronic obstructive pulmonary disease (AECOPD). The Modified DECAF score, which includes exacerbation frequency as an additional parameter, resulted in reclassification of several patients into higher severity categories compared to the original DECAF scoring system. Chi-square test results are presented below the table.

Score	DECAF Score (n)	DECAF Score (%)	Modified DECAF Score (n)	Modified DECAF Score (%)
0	47	92.20%	5	9.80%
1	4	7.80%	42	82.40%
2	0	0.00%	4	7.80%

Correlation between scores

To assess consistency between the DECAF and Modified DECAF scores, Spearman’s rank correlation was applied. A strong positive correlation was observed between the two scores (ρ = 0.702, p < 0.00001), indicating that as DECAF scores increase, modified DECAF scores tend to increase proportionally (Table 4). This finding supports the modified DECAF as an extension of the original scoring model rather than a conflicting or divergent tool. The results of Spearman’s rank correlation test indicate a strong positive correlation (ρ = 0.702, p < 0.00001) between DECAF and Modified DECAF scores (Table 4). Table 5 presents agreement analysis using Cohen’s Kappa and evaluates classification shifts using McNemar’s test. A Kappa coefficient of 0.00 suggests no agreement beyond chance between the two systems for identifying high-risk patients. Although four patients (n = 4, 7.8%) were reclassified as high risk by the modified DECAF, this change was not statistically significant (p = 0.125). 

**Table 4:** Spearman Correlation Between DECAF and Modified DECAF Scores Spearman’s rank correlation revealed a strong positive correlation (ρ = 0.702, p < 0.00001), indicating that increases in Dyspnea, Eosinopenia, Consolidation, Acidemia, and Atrial Fibrillation (DECAF) scores were associated with proportional increases in Modified DECAF scores.

Statistical Test	Correlation Coefficient (ρ)	p-value
Spearman Correlation	0.702	<0.00001

**Table 5:** Agreement and Classification Shift Between DECAF and Modified DECAF Risk Stratification Systems Agreement analysis between Dyspnea, Eosinopenia, Consolidation, Acidemia, and Atrial Fibrillation (DECAF) and Modified DECAF scores in risk stratification. Cohen’s Kappa coefficient showed no agreement beyond chance, and McNemar’s test confirmed that the reclassification was not statistically significant. Degrees of freedom and effect size (ϕ) are reported to quantify the strength of association.

Comparison	Kappa Coefficient (κ)	Agreement Level
DECAF vs Modified DECAF Risk	0	No agreement beyond chance
DECAF vs Modified DECAF	Modified Low	Modified High
DECAF Low	47	0
DECAF High	4	0
McNemar p-value		0.125

Agreement in risk classification

Despite the strong correlation, categorical agreement between the DECAF and Modified DECAF scores was low. Cohen’s Kappa coefficient revealed no agreement beyond chance (κ = 0.00) in risk stratification. McNemar’s test indicated that the reclassification of four patients (n = 4, 7.8%) from DECAF low risk to Modified DECAF high risk, based on exacerbation frequency, was not statistically significant (χ²(1, N = 51) = 2.25, p = 0.125). The effect size, measured using the Phi coefficient (ϕ = 0.210), suggests a small association between the scoring systems (Table 4).

Statistical significance of reclassification

To evaluate whether this reclassification was statistically significant, McNemar’s test was conducted. Although a shift was noted (from DECAF low risk to Modified DECAF high risk in 4 patients, n = 4, 7.8%), the result was not statistically significant (p = 0.125) (Table 5). Nonetheless, the trend emphasizes that modified DECAF may offer incremental clinical insight by identifying patients with an otherwise low DECAF score but a burdensome exacerbation history. Table 5 presents the agreement analysis between DECAF and Modified DECAF scores using Cohen’s Kappa and evaluates classification shifts using McNemar’s test. A Kappa coefficient of 0.00 suggests no agreement beyond chance between the two systems for identifying high-risk patients. The modified DECAF reclassified four patients as high risk. However, this change was not statistically significant (McNemar’s χ²(1) = 2.25, p = 0.125).

**Figure 1:**
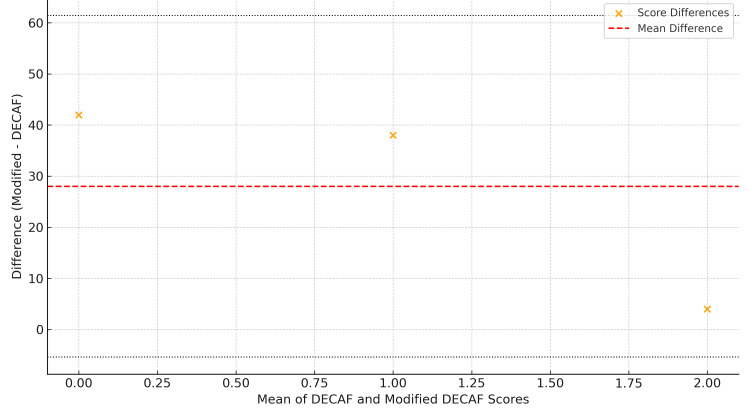
Bland-Altman Plot Showing Agreement Between DECAF and Modified DECAF Scores This plot displays the mean score versus the difference between Dyspnea, Eosinopenia, Consolidation, Acidemia, and Atrial Fibrillation (DECAF) and Modified DECAF scores across score levels. The red dashed line indicates the mean difference, with dotted lines marking the 95% limits of agreement. The visualization underscores the redistribution of scores while highlighting the overall alignment between both scoring systems.

To evaluate the agreement between the original DECAF and the Modified DECAF scores, a Bland-Altman analysis was performed. The plot (Figure 1) illustrates the distribution of score differences against the mean of the two scores. Most values cluster closely around the mean, suggesting good agreement, despite some reclassification observed between scoring systems. The mean difference was modest, indicating that while the modified DECAF score shifts some patients into different risk categories, the overall concordance with the original DECAF is preserved. No statistically significant associations between risk reclassification and clinical outcomes (such as mortality, ICU admission, or length of stay) could be firmly established due to limited events and sample size.

While the modified DECAF score identified four additional patients as high risk based on exacerbation frequency, this reclassification was not statistically significant, and no improvement in clinical outcomes (e.g., mortality or ICU admission) could be demonstrated within this cohort. These findings should be interpreted with caution in light of the study’s retrospective, single-center design, limited sample size, and absence of external validation or outcome analysis. No analyses of subgroups were possible due to small numbers, and the accuracy of exacerbation history may be influenced by the quality of available medical records. The observed strong correlation between scores suggests that the modified DECAF performs largely in parallel with the original tool; however, whether its higher sensitivity will translate into meaningful improvements in patient triage or management requires validation in larger, multicenter prospective studies. In summary, while the modified DECAF score demonstrates a strong statistical correlation with the original and a greater tendency to identify patients with frequent exacerbations as higher risk, these changes were not statistically significant and did not associate with measurable differences in clinical outcomes within this limited, retrospective cohort.

## Discussion

The present study aimed to evaluate the correlation and agreement between the DECAF and Modified DECAF scores in assessing severity among patients with AECOPD. Our findings demonstrate a strong positive correlation (ρ = 0.702, p < 0.00001) between the two scoring systems; however, categorical agreement was lacking (κ = 0.00), particularly after the inclusion of exacerbation frequency in the modified version. This modification appears to enhance risk stratification by capturing clinically relevant patient subsets that may be overlooked by the original DECAF score.

This aligns with the observations of Grolimund et al., who emphasized that exacerbation frequency, clinical biomarkers, and exacerbation phenotype significantly affect long-term prognosis in COPD [9]. Similarly, Darshini et al. reported that the Modified DECAF score was superior to the COPD Assessment Test (CAT) in predicting ICU admission and hospital outcomes, suggesting greater clinical applicability [10]. Adinarayan et al. further established that the Modified DECAF score offers improved prognostic accuracy compared to neutrophil-lymphocyte and platelet-lymphocyte ratios, especially in predicting mortality and hospital stay in AECOPD patients [11].

In our cohort, 92.2% of patients had a DECAF score of 0, classifying them as low risk. However, the modified DECAF reclassified 90.2% of these patients into higher-risk categories, an observation consistent with previous studies emphasizing the need for more sensitive stratification tools. The high rate of reclassification to higher-risk categories by the modified DECAF score in our cohort underscores its greater sensitivity in identifying patients with less overt but clinically important risk factors, such as frequent exacerbations. This suggests that the modified DECAF may reveal a subset of patients who, despite appearing low risk on the original DECAF, have a more active and unstable disease profile, warranting closer monitoring or intensified management. In practice, such reclassification could prompt interventions like increased frequency of clinical assessments, earlier involvement of multidisciplinary teams, or proactive planning for follow-up after discharge, all of which may help prevent further exacerbations and hospitalizations. From a healthcare system perspective, adopting a more sensitive risk stratification tool could also support more effective allocation of clinical resources by ensuring that high-risk patients receive timely interventions. However, these benefits must be balanced against the potential for overtreatment, unnecessary resource utilization, and increased patient anxiety if heightened sensitivity comes with reduced specificity. Therefore, while our findings and the consistent positive bias observed in the Bland-Altman analysis support the value of the modified DECAF in drawing attention to patients who may otherwise be underestimated, further research on larger samples is needed to determine if this reclassification results in improved long-term outcomes or merely greater awareness of risk [10,11]. While McNemar’s test did not show statistical significance (p = 0.125), indicating that reclassification may not translate into clear categorical shifts in smaller samples, the Bland-Altman analysis (Figure 1) revealed a consistent positive bias, demonstrating the Modified DECAF's tendency to assign higher scores.

This observation supports the findings of Shen et al., whose meta-analysis validated the diagnostic accuracy of the DECAF score in various clinical contexts [12]. Furthermore, Gulcan et al. showed that augmenting DECAF with additional biochemical markers like lactate improved mortality prediction while maintaining clinical usability [13]. International validation by Almarshoodi et al. reinforced the adaptability and robustness of the DECAF score across diverse populations and healthcare settings [14], while Choudhary et al. reported that incorporating frequent exacerbations into the DECAF framework enhanced its prognostic reliability in Indian cohorts [15].

The broader utility of the DECAF scoring system in predicting short-term mortality, particularly in elderly populations, was emphasized by Ergene et al. and Ergül et al., who both demonstrated its superior prognostic accuracy compared to other risk models in emergency department settings [16-18]. These insights further strengthen the rationale for localized or context-specific modifications of the DECAF score, such as the one tested in our study. The incorporation of exacerbation frequency aligns with established GOLD guidelines, reflecting the recognized importance of recent exacerbations in risk stratification for COPD. However, our definition was based solely on the number of medically attended events over the prior year; we did not further classify exacerbations by severity or require documentation of ICU admission or intubation for this parameter. This limited granularity may affect risk assessment and should be addressed in future studies. Although the modified DECAF score reclassified a small percentage (7.8%) of patients into a higher-risk category, this shift did not reach statistical significance, nor was it supported by corresponding changes in clinical outcomes such as mortality or ICU admission in our cohort. Therefore, our findings do not demonstrate a clear predictive improvement of the modified DECAF over the original score. Any assertion of enhanced clinical utility should be interpreted with caution and awaits confirmation in future studies with larger sample sizes and robust outcome data. This study's retrospective design introduces the risk of information bias, particularly in exacerbation history ascertainment. The lack of a formal power calculation, small sample size, potential for missing data, and absence of external or long-term mortality validation should be considered when interpreting findings. The statistical limitations of Cohen’s Kappa in skewed datasets and single-center design may further affect generalizability. Future research should include prospective, multicentric validation with direct outcome measurement and a more detailed definition/ascertainment of exacerbation frequency.

## Conclusions

The present study demonstrates a strong positive correlation between the DECAF and modified DECAF scores in assessing severity among AECOPD patients. Notably, the lack of agreement in risk categorization reflects the modified DECAF score’s ability to identify additional patients at higher risk-particularly those with frequent exacerbations. Although the numerical reclassification did not reach statistical significance in our analysis, the modified DECAF shows potential for enhanced sensitivity in clinical risk stratification, which may ultimately help to guide more tailored management, especially in resource-limited settings.

However, these findings must be interpreted in light of certain limitations, including the study’s small sample size and retrospective, single-center design, which may limit the generalizability and robustness of our results. Larger, prospective, multi-center studies are warranted to validate the clinical utility and prognostic superiority of the modified DECAF score, and to determine whether its use translates into better patient outcomes.
